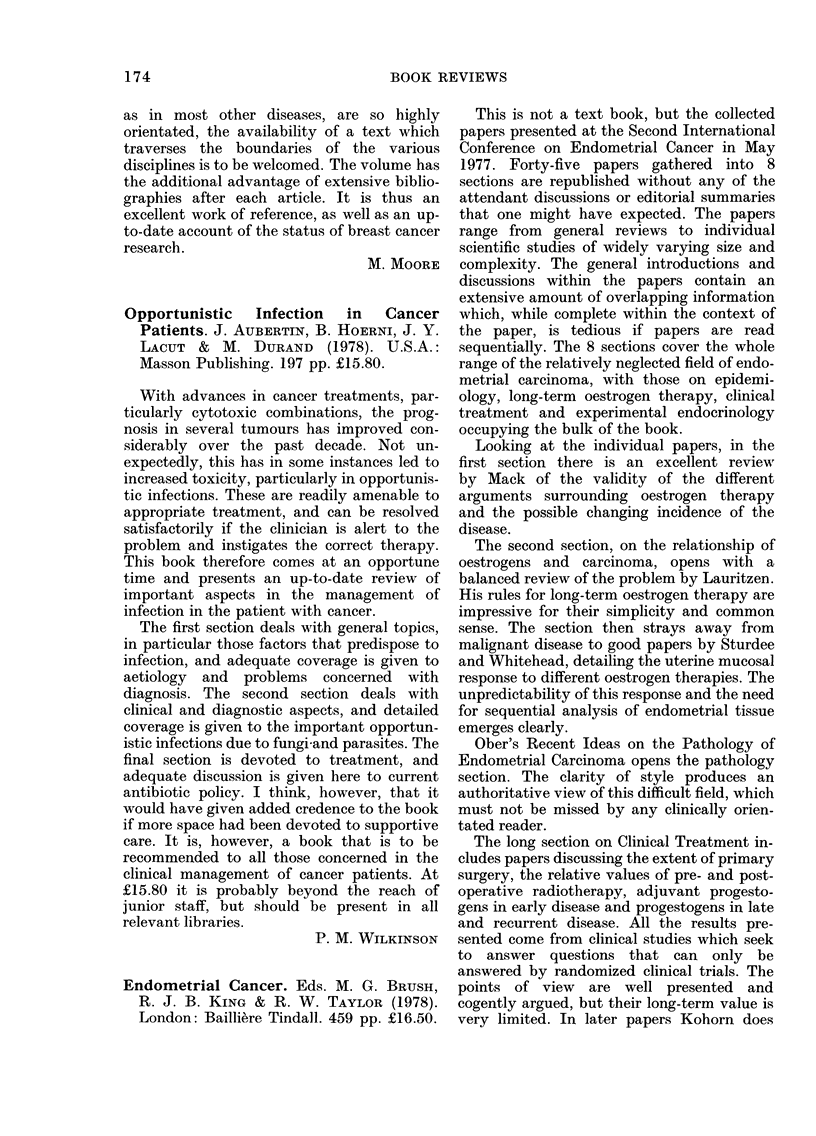# Opportunistic Infection in Cancer Patients

**Published:** 1979-07

**Authors:** P. M. Wilkinson


**Opportunistic Infection in Cancer Patients**. J. AUBERTIN, B. HOERNI, J. Y. LACUT & M. DURAND (1978). U.S.A.: Masson Publishing. 197 pp. £15.80.

With advances in cancer treatments, particularly cytotoxic combinations, the prognosis in several tumours has improved considerably over the past decade. Not unexpectedly, this has in some instances led to increased toxicity, particularly in opportunistic infections. These are readily amenable to appropriate treatment, and can be resolved satisfactorily if the clinician is alert to the problem and instigates the correct therapy. This book therefore comes at an opportune time and presents an up-to-date review of important aspects in the management of infection in the patient with cancer.

The first section deals with general topics, in particular those factors that predispose to infection, and adequate coverage is given to aetiology and problems concerned with diagnosis. The second section deals with clinical and diagnostic aspects, and detailed coverage is given to the important opportunistic infections due to fungi and parasites. The final section is devoted to treatment, and adequate discussion is given here to current antibiotic policy. I think, however, that it would have given added credence to the book if more space had been devoted to supportive care. It is, however, a book that is to be recommended to all those concerned in the clinical management of cancer patients. At £15.80 it is probably beyond the reach of junior staff, but should be present in all relevant libraries.

P. M. WILKINSON